# DNA Barcoding of Recently Diverged Species: Relative Performance of Matching Methods

**DOI:** 10.1371/journal.pone.0030490

**Published:** 2012-01-17

**Authors:** Robin van Velzen, Emanuel Weitschek, Giovanni Felici, Freek T. Bakker

**Affiliations:** 1 Biosystematics Group, Wageningen University, Wageningen, The Netherlands; 2 Netherlands Centre for Biodiversity Naturalis, Section NHN – Wageningen Branch, Wageningen University, Wageningen, The Netherlands; 3 Istituto di Analisi dei Sistemi e Informatica Antonio Ruberti, Consiglio Nazionale delle Ricerche, Rome, Italy; 4 Department of Informatics and Automation, University Roma Tre, Rome, Italy; University of Vermont, United States of America

## Abstract

Recently diverged species are challenging for identification, yet they are frequently of special interest scientifically as well as from a regulatory perspective. DNA barcoding has proven instrumental in species identification, especially in insects and vertebrates, but for the identification of recently diverged species it has been reported to be problematic in some cases. Problems are mostly due to incomplete lineage sorting or simply lack of a ‘barcode gap’ and probably related to large effective population size and/or low mutation rate. Our objective was to compare six methods in their ability to correctly identify recently diverged species with DNA barcodes: neighbor joining and parsimony (both tree-based), nearest neighbor and BLAST (similarity-based), and the diagnostic methods DNA-BAR, and BLOG. We analyzed simulated data assuming three different effective population sizes as well as three selected empirical data sets from published studies. Results show, as expected, that success rates are significantly lower for recently diverged species (∼75%) than for older species (∼97%) (P<0.00001). Similarity-based and diagnostic methods significantly outperform tree-based methods, when applied to simulated DNA barcode data (P<0.00001). The diagnostic method BLOG had highest correct query identification rate based on simulated (86.2%) as well as empirical data (93.1%), indicating that it is a consistently better method overall. Another advantage of BLOG is that it offers species-level information that can be used outside the realm of DNA barcoding, for instance in species description or molecular detection assays. Even though we can confirm that identification success based on DNA barcoding is generally high in our data, recently diverged species remain difficult to identify. Nevertheless, our results contribute to improved solutions for their accurate identification.

## Introduction

Recently diverged species are frequently of special interest, for example in ecology, regulation or forensics [1], [2], [3], and hence their accurate identification is warranted. DNA barcoding [4], [5], [6], [7] has proven instrumental in identifying recently diverged species (e.g. species complexes or cryptic species) that are of importance to conservation biology [8], [9], [10], [11], pest management [12], [13], [14], fishery [15], [16], [17], [18], [19], [20], invasive biology [21], [22], [23], [24], [25], [26] and disease control [27], [28], [29], [30]. In some cases, however, identification of recently diverged species using DNA barcodes has been reported to be problematic [1], [2], [31], [32], [33], [34] due to ambiguous barcode matches or the absence of barcode clusters in DNA barcode trees.

Failure of DNA barcodes to properly resolve recently-diverged species can be attributed to population genetic factors of the species involved [31], [35], [36], [37], [38], [39]. Coalescent theory [40] predicts that the chance that gene sequences sampled from a species are monophyletic is dependent on the age of that species (measured in number of generations since speciation) and reversely dependent on its effective population size (*N_e_*) [40], [41]. This is because species with large *N_e_* are predicted to have larger within-species genetic variation [40], [41], [42]. When such species have diverged only recently their gene sequences are likely to have a most recent common ancestor predating the speciation event (incomplete lineage sorting) [42]. This results in overlapping within- and between-species genetic distances (lack of a ‘barcode gap’) and paraphyly or even polyphyly of conspecific samples in gene trees [42], [43], [44], [45]. For example, in Lycaenidae (Blue butterflies) Wiemers and Fiedler [38] found a general lack of ‘barcode gaps’ and paraphyly or polyphyly of conspecific DNA sequences, probably caused by incomplete lineage sorting [38], as did McFadden *et al.* in Octocorals [36]. Meyer and Paulay [31], in their DNA barcode study of marine gastropods, explained non-monophyly of some species by incomplete lineage sorting effects. Elias *et al.* [43] reported limited performance of DNA barcoding in two butterfly communities in Ecuador, which they attributed in part to large *N_e_* and associated long coalescent times [39]. Based on simulated DNA barcode data sets Ross *et al.* [37] and Austerlitz *et al.* [35] found that species monophyly and identification success generally decreased with increasing coalescent depth.

Regardless of *N_e_*, recently diverged species have acquired only few genetic differences meaning that there are few characters to discriminate them. The rate at which two sister species genetically diversify is dependent on their effective mutation rate (µ). If µ is sufficiently low, even reciprocally monophyletic species will share identical haplotypes. Indeed, some morphologically well-differentiated species may share identical DNA barcode sequences, preventing accurate identification using DNA barcodes [33], [36], [38]. If µ is higher, identification success depends on the extent of lineage sorting: on the one hand, a single fixed mutation can be enough for successful identification [36], [46], [47]; on the other hand, non-monophyletic (i.e. incompletely-sorted) species will have overlapping genetic variation even when µ is high. Therefore, we consider the factors governing lineage sorting: time (measured in generations), and *N_e_,* to be the most important factors contributing to DNA barcode identification problems with recently diverged species. Obviously, when given enough time any *N_e_* or µ will ultimately result in high levels of between-species divergence. We therefore emphasize time here and focus on ‘recent’ versus ‘old’ species.

Various methods have been proposed to match DNA barcodes to a reference library for identification, amongst which we recognize the following:

**Tree-based** methods assign unidentified (query) barcodes to species based on their membership of clusters (or clades) in a DNA barcode tree. This approach is usually based on neighbor joining [48], [49], parsimony [50] or Bayesian inference [51]. Tree-based methods assume that samples of distinct species form discrete clusters in a DNA barcode tree [4], [49]. It is generally acknowledged, however, that gene trees (i.e. DNA barcode trees) do not necessarily reflect organismal history [42], and that the incomplete lineage sorting effects outlined above may lead to incorrect identifications based on such trees [31], [35], [37], [38], [39].

**Figure 1:**
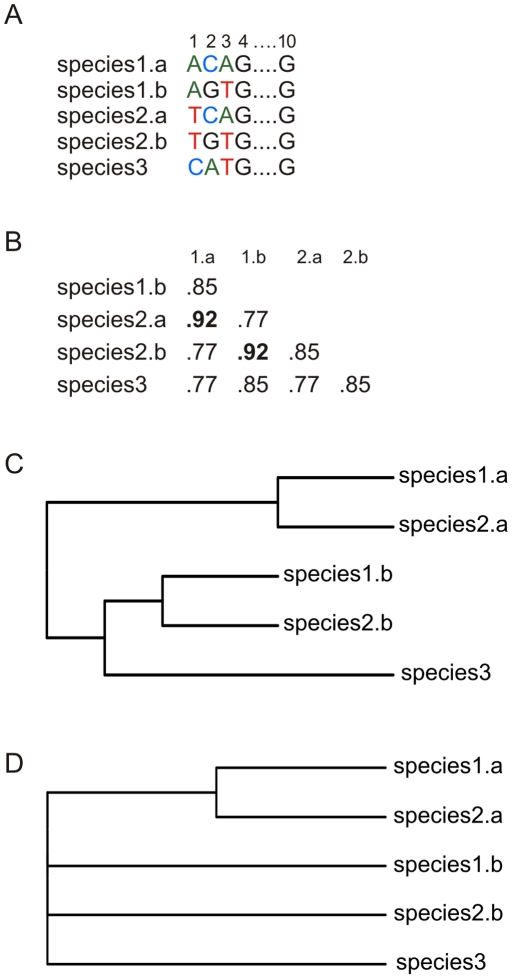
Hypothetical DNA barcode sequences where tree-based and similarity methods produce incorrect identifications. A. Alignment where two recently diverged sister species (species1 and species2) have only one diagnostic nucleotide differentiating them from each other (position 1) and at the same time share two polymorphisms (positions 2 and 3). Species3 is included as outgroup; B. Pairwise uncorrected similarities based on the alignment with highest pairwise similarities in boldface; C. Neighbor joining tree; D. Strict consensus of all maximum parsimony trees.

**Similarity-based** methods assign query barcodes to species based on how much DNA barcode characters they have in common. Similarity can be calculated directly from nucleotide sites (e.g. using MOTU [52], nearest neighbor [35], [53], or BLAST [54]) or from a projection of nucleotides (e.g. Kernel methods [35], [55], [56], ATIM [57], BRONX [58]). Similarity-based methods assume that conspecific samples will be more similar to each other than to samples of any other species. However, this need not be true in all cases. For instance, if we consider two hypothetical sister species that share two polymorphisms and have only one nucleotide differentiating them from each other, tree- and similarity-based methods will fail to correctly identify (some of the) haplotypes in these species, see Figure 1.

**Statistical** methods estimate confidence measures on DNA barcode matches for species identification. These methods typically employ Bayesian estimation based on explicit population genetic or phylogenetic models [44], [59], [60]. Obviously, confidence measures are of great importance when dealing with regulated species, forensics or disease vectors [44]. However, because statistical methods for species identification are computationally intensive and the appropriate model parameters are not known for the majority of species we will not treat them further.

**Diagnostic** methods (sometimes included in ‘character-based’ methods [46]) rely on the presence/absence of particular characters in DNA barcode sequences for identification, instead of using them all. Diagnostics can be either “simple” when based on a single unique character or “compound” when based on a unique combination of characters [61]. Some methods use nucleotide data and require a multiple sequence alignment (e.g. CAOS [61], [62], [63], BLOG [64]. Others use diagnostic nucleotide strings as diagnostics and are therefore alignment-free (e.g. DNA-BAR [65]). Diagnostic methods are analogous to classical taxonomic practices that rely on morphological diagnostic characters [46], [66]. As opposed to other methods, diagnostic methods have the potential to select the differentiating nucleotide only and ignore any within-species variation obscuring that signal [46], [67], [68]. For example, a diagnostic method could correctly identify the two hypothetical species in Figure 1 based on the diagnostic nucleotide at position 1.

Our objective was to compare relative performance of six DNA barcode matching methods in correctly identifying barcodes of recently diverged species. Below we provide some motivations for choosing each of these six methods:

1. Tree-based neighbor joining (NJ) [48] because it is the most widely used method for classifying DNA barcodes in the literature, and implemented in, for instance, the Barcode Of Life Database [69]. Speed being its main advantage, NJ is a bottom-up clustering algorithm that calculates a single tree from a distance matrix. Results can be dependent on the ordering of the matrix, however, making results sometimes less reproducible. The underlying assumption in NJ barcode matching is that barcode sequences of distinct species form discrete clusters in a NJ tree [4]. For identification, query sequences are included in the NJ tree to see in which cluster they appear.
2. Tree-based parsimony (PAR) [50] as it outperformed other tree-based methods (such as the Statistical Assignment Package SAP [49]), in a published comparative study [58]. PAR adopts the optimality criterion under which the preferred tree is the tree that requires the least evolutionary change to explain the data. Assessing all possible trees for more than 20 sequences is computationally impossible and therefore PAR methods employ heuristics to find the preferred tree(s).
3. Similarity-based nearest neighbor (NN) because it gave high correct identification rates in previous studies [35], [53]. Based on a distance matrix, NN simply assigns a query sequence to the same species membership as its closest sequence in the reference data base. It is equivalent to the ‘Best Match’ method by Meier *et al.* [53] and the ‘1-NN’ method used by Austerlitz *et al.* [35].
4. Similarity-based BLAST [54] as it is probably the most commonly used method for classifying DNA sequences in practice. It is an algorithm for comparing query sequences with an unaligned reference data base calculating pairwise alignments in the process. It is faster than NN, but can give incorrect matches in some cases, especially with incomplete reference data bases [70].
5. The diagnostic method DNA-BAR [65] because it showed higher levels of accurate species identification in previous studies [57], [58] compared to the other diagnostic method CAOS [61]. DNA-BAR first selects sequence substrings (distinguishers) differentiating the sequences in the reference data set, and then records presence/absence of these distinguishers. An advantage of using substrings is that the method does not require an alignment.
6. The recently developed diagnostic logic mining method BLOG [64] because it has not been used in any comparative test before (except [71]). BLOG first selects a number of characters (‘features’) from the reference data set that optimize discrimination of a particular species, based on an integer programming feature selection method. It then uses the selected features to search for the simplest logic formula that discriminates that species from all others using a learning method based on decomposition techniques [64], [71]. This process is reiterated for every species in the reference data set. Subsequently, query sequences are screened for their recognition by the formulas for identification. The reader may refer to [64], [72], [73] and [74] for a complete description of the mathematical models that constitute the main characteristics of BLOG.

We use simulated and empirical DNA barcode datasets, the latter from published studies. In general, data simulations allow for replication and, hence, statistical testing of method performance. For instance, Austerlitz *et al.* [35] assessed relative performance of NJ, NN, classification and regression trees, random forest, and kernel methods in correctly assigning query barcodes to predefined species. They concluded that, although NN was the most reliable method overall, none was found to be best under all circumstances. However, the authors simulated datasets with only 2–5 species and assumed simultaneous divergence of all species which seems biologically unrealistic [35]. Here, we simulated more realistic DNA barcode datasets comprising 50 species along a phylogenetic tree, thus producing more typical levels of sequence divergence. In this regard our approach is similar to that of Ross *et al.* [37] who tested similarity and tree-based methods of species identification using ‘realistic’ simulated datasets. They concluded that tree-based methods returned ambiguous identifications. However, they did not take species divergence times explicitly into account, nor did they include diagnostic methods, which we do here.

Our results show that, even though recently diverged species pose a significant problem for effective DNA barcoding, sensitive similarity-based and diagnostic methods can significantly improve identification performance compared with the commonly used tree-based methods such as NJ.

## Materials and Methods

Our analytical pipeline started with generating simulated DNA barcode data sets and selection of published empirical data sets. Subsequently, we assessed both ‘barcode gap’ and monophyly of species and performed matching analyses with tree-based (NJ,PAR), similarity-based (NN, BLAST) and diagnostic (DNA-BAR, BLOG) methods on both types of data. The pipeline concluded with a comparative evaluation of methods used in terms of accuracy of species identification.

### Data simulation

DNA barcode datasets were simulated using the Coalescent package in Mesquite version 2.73 build 544 [75], [76]. We simulated along two axes: time of species divergence and effective population size (*N_e_*). We started by simulating a random ultrametric species tree for 50 species using the Yule model [77], with a total tree depth of 1 million generations. Species were divided into two equally-sized groups (N = 25) based on their rank in divergence times: one with ‘recently diverged’ species and another with ‘old’ species.

Ultrametric gene trees were simulated on the ultrametric species tree according to the coalescence model, generating 20 individuals per species. Gene trees were simulated using *N_e_* = 1,000 10,000 and 50,000 with each simulation replicated 100-fold, resulting in 300 gene trees in total. Additive gene trees were then obtained by adding noise to the branch lengths of gene trees in order to ensure more realistic (i.e. non-ultrametric) data structure. Thereby we effectively mimicked heterogeneity of the effective mutation rate (µ) over branches of the gene trees. Noise was normally distributed, with a variance *σ* of 0.7 times the original branch length.

DNA barcode sequences were then simulated on the additive gene trees according to a HKY substitution model [78], the choice of which was based on the best-fitting model for a representative empirical dataset of 527 Nymphalidae DNA barcodes as selected using JModelTest 0.1.1 [79] applying the AIC criterion. Model parameters encompassed a transition/transversion ratio *κ* of 8.3, nucleotide frequencies of 0.30 (A), 0.15 (C), 0.10 (G), 0.45 (T), and gamma-distributed rate variation over sites with 4 rate categories and a shape parameter *α* of 0.2. Sequence length was 650 base pairs, approximating the length of the standard DNA barcode for animals (*COI*). Simulated sequences were divided over reference data sets (16 sequences per species) and query data sets (4 sequences per species). The reference data sets were considered as DNA barcode reference libraries containing sequences with *a priori* assigned species membership. The query data sets were considered to comprise unknown DNA barcodes, although in our case species membership was known because they were simulated together with the reference data set. Consequently, accuracy of their identification could be evaluated *a posteriori*.

### Empirical data sets

We selected three published empirical DNA barcode data sets based on the following criteria: 1. Data contain species that are problematic to identify using DNA barcodes because of incomplete clustering in barcode trees; 2. Data encompass high phylogenetic diversity, i.e. from different phyla (Plantae, Mollusca and Arthropoda), to ensure the general applicability of our outcomes; 3. Data come from different markers, i.e. from all three genomic compartments. A summary of the selected data can be found in Table 1; details are below:

**Table 1:** Summary of selected empirical data sets used.

Data set	ref.	marker	seq. length	#sequences	#spp.	#spp. ≥ 5 seq.
*Drosophila*	[33]	*COI*	663	615	19	15
*Inga*	[1]	*trnTD*, ITS	1838	913	56	35
Cypraeidae	[31]	*COI*	614	2008	211	112

Ref. =  Reference to original publication, seq.length =  sequence length, #sequences  =  number of sequences, #spp.  =  total number of species in the data set, #spp. ≥ 5 seq.  =  number of species represented by 5 or more sequences.

***Drosophila*** **.** Lou and Golding [33] used this data set to test the ability of algorithms to assign sequences to species in the absence of a barcode gap. They found that many species are siblings with low between-species distances and some have no ‘barcode gap’ [23], [33]. *Drosophila* species are also known to have relatively large *N_e_*’s and associated high within-species divergence [80], [81]. The data set comprised 615 barcodes from 19 species.

***Inga*** (Fabaceae) is a large genus of tropical leguminous trees. Many morphologically distinct *Inga* species collected in the southwestern Amazon are incompletely sorted in DNA barcode trees [1]. No *N_e_* estimates for *Inga* are available. We selected the data set from Dexter *et al.* [1] who linked cpDNA *trnTD* intron and nrDNA Internally Transcribed Spacer (ITS) sequences into a multi-locus DNA barcode of 1713–1771 nucleotides in total. The data set comprised 913 barcodes from 56 species

**Cypraeidae** (Mollusca) are taxonomically one of the most extensively studied marine gastropods. Although Meyer & Paulay showed that subspecies rather than species best represent diversity in these DNA barcodes [31] we adhered to species names, mainly because subspecies were generally less well sampled. No *N_e_* estimates for Cypraeidae are available. The data set comprised 2008 mtDNA COI sequences of 211 species and had almost complete coverage of sister-species, some of which are reported to have diverged only recently [31].

Only those species represented by 5 or more sequences were evaluated in the identification assessments. Their sequences were randomly distributed over a reference data set (80% per species) and a query data set (20% per species). Species represented by less than 5 sequences were kept in the reference data set, but not evaluated in the identification assessments (i.e. their sequences could therefore only contribute to the false positive rate of the query sequences that were evaluated).

### Species ‘barcode gap’ and monophyly

To assess the existence of a ‘barcode gap’ in our data sets, we extracted within- and between- species K2P [82] distances from all 50 species in all 300 simulated reference data sets (100 of each *N*_e_) and made comparisons between *N_e_*'s. We are aware that using K2P implies effective under-parameterization [83] as we used HKY in the simulations, but we chose K2P as it is typically used in DNA barcode analyses (e.g. [10], [15], [17], [25], [84]). Repeating the analysis using HKY did not give different results (not shown). We evaluated the existence of ‘barcode gaps’ at species level by scoring a species as having a ‘barcode gap’ when the minimum between-species sequence distance exceeded the maximum within-species distance [31], [85].

We assessed species-monophyly in DNA barcode trees of all 50 species in all 300 simulated reference data sets and subsequently compared results between *N_e_*'s. DNA barcode trees were reconstructed using NJ and parsimony using settings described below, and species were scored as either monophyletic or non-monophyletic based on the DNA barcode tree topologies.

### Method performance

#### Neighbor joining (NJ)

We used the neighbor joining algorithm [48] implemented in the R. package APE 2.5–3 [86] and applied randomly shuffling of input order of sequences. We assessed tree topology in two ways, following Ross *et al.* [37]. 1. ‘Strict assessment’ meant that if the query was nested within a mono-specific cluster or clade it was identified as that species. Otherwise its identification was considered uncertain. This is equivalent to the ‘Tree based identification, revised criteria’ used by Meier *et al.* [53] and is reported to have significantly lower false-positive rates [37]. 2. ‘Liberal assessment’ meant that if the query was sister to a mono-specific cluster it was identified as that species. Otherwise its identification was considered uncertain.

#### Parsimony (PAR)

Maximum parsimony trees were estimated using TNT version 1.1 [87]. Heuristic searches consisted of iterations of ratchet, sectorial searches, tree drift and tree fusing algorithms [88] through the TNT built-in function ‘xmult’, holding 1000 trees during search (‘hold 1000’). Searches were stopped when four independent replicates found shortest trees of the same length (‘xmult =  hits 4’). Identical sequences were excluded before analysis and later restored to save computation time (‘riddup’). Only one maximum parsimony tree was held after each analysis to make results comparable to NJ. We assessed tree topology in the same way as described for NJ above.

#### Nearest neighbor (NN)

Nearest neighbors were calculated using the ‘dist.dna’ function in the R. package APE version 2.5–3 [86] based on the K2P model of sequence evolution [82]. A query was identified as the species associated with its nearest neighbor (reference sequence with lowest distance to that query). In case nearest neighbors were from more than one species the query's identification was considered uncertain.

#### BLAST

Identification based on BLAST was performed using NCBI software version 2.2.25+ [89]. Reference data sets were stored in a BLAST database for subsequent matching with query sequences. Up to 100 hits with at least 80% identity were returned for each query, which was identified as the species associated with its best hit (highest bit score). In case more than one species were associated the query's identification was considered uncertain.

#### DNA-BAR

Reference data sets were converted to a matrix comprising presence/absence of distinguishers (sequence substrings) using the software ‘degenbar’ [65]. Input parameters were as follows: distinguishers of length 5–50 nucleotides (‘l-min 5’, ‘l-max 50’), up to 100 redundant distinguishers (‘Redundancy 100’), GC content 0–100% (‘MinCandidGC 0’, ‘MaxCandidGC 100’), annealing temperature 0–100°C (‘MinCandidTemp 0’, ‘MaxCandidTemp 100’), salt and DNA concentration 50 nM (‘SaltConc 50’, ‘DNAconc 50’), and a maximum common substring weight of 100 (‘MaxCommSubstrWt 100’) (note that degenbar was originally designed to pick DNA probes). In case of multi-locus DNA barcodes (i.e. *Inga* data set) loci in the reference alignment were separated by 50 ‘N’ positions. The presence/absence matrix of distinguishers was then used as reference data set. Each query sequence was scored for presence/absence of distinguishers and identified as the species associated with the reference sequence with the greatest number of matching presence/absences. In case more than one reference sequence of the same species membership shared the greatest number of matches the query was identified as that species. In case reference sequences associated with different species shared the greatest number of matches identification was considered uncertain.

#### BLOG

Diagnostic logic mining analyses were performed with BLOG software version 2.4 [64] which is available online [90] and on the Barcode Of Life Data Portal [91], [92] (an off-line version is available from EW upon request). Input parameters for feature selection were as follows: a maximum number of 35 features chosen (‘BETA = 35’), a maximum of 200 iterations (‘GRASPITER  = 200’), and a maximum time of 500 minutes for analysis (‘GRASPSECS = 30000’). Each query sequence was scanned to see if it satisfied any of the logic formulas generated by BLOG and identified as the species associated with the matching logic formula. In case a query satisfied more than one logic formula the logic formula having lowest false positive rate on the reference data set was taken as the identification. In case error rates of logic formulas were equal identification was considered uncertain.

### Statistical tests

We assessed relative performance of the six methods in terms of their identification success with simulated and empirical data. Identification success was defined in two ways: 1. ‘Species identification success’ was scored as the number of species for which all query sequences were correctly identified. 2. ‘Sequence identification success’ was scored as the number of correctly identified query sequences per data set, which is equivalent to sensitivity (i.e. true positives/[true positives + false negatives]).

We evaluated the influence of i) species divergence times (recently diverged versus old), ii) method used, and iii) *N_e_* on species identification success, using Friedman tests [93] in which the sum of identification success measures per replicate was used as the observation. Significant differences between methods were revealed in post-hoc pairwise Wilcoxon signed rank tests based on paired observations [94]. To account for the large number of comparisons we applied Bonferroni correction [95] to all tests combined (i.e. multiplying p-values by total number of tests performed). A corrected value of P<0.01 was considered statistically significant.

## Results

### Data simulation

The 50 species in the simulated ultrametric species tree had divergence times between 98 and 553116 generations (see Figure S1). We classified half the species (with divergence times between 98 and 76621 generations) as ‘recently diverged’ and the other half (with divergence times between 76621 and 553116 generations) as ‘old’, see figure S1.

### Species ‘barcode gap’ and monophyly

**Figure 2:**
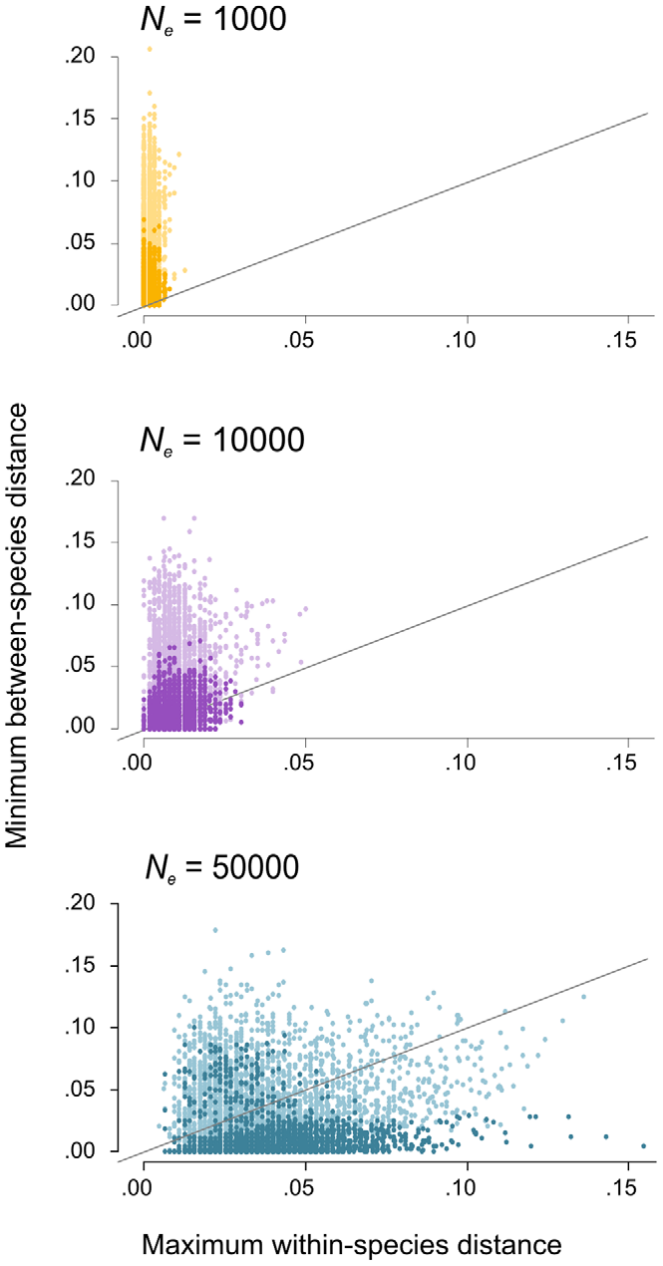
Species ‘barcode gap’. Scatterplots of minimum between- over maximum within-species distance for 5000 simulated species in the reference data sets with 16 samples per species. Simulations under coalescence with effective population sizes (*N_e_*) of 1000 (yellow, top), 10000 (purple, middle) and 50000 (blue, down) individuals. Brightness of the dots correlates with species divergence times, i.e. recently diverged species are dark and old species are light. Species plotted above the diagonal lines have a barcode gap.

Maximum within-species distance equals or exceeded minimum between species distance for a substantial proportion (37%) of the species in the simulated data sets, indicating absence of a barcode gap. This proportion positively correlates with effective population size (*N_e_*), which is explained mainly by an increase of the within-species distances under larger *N_e_*, see Figure 2. On the contrary, with 54% for old species and 20% for recently diverged species this proportion decreases with increasing divergence time (mostly dark dots fall below the ‘barcode gap’ line in Figure 2).

**Figure 3:**
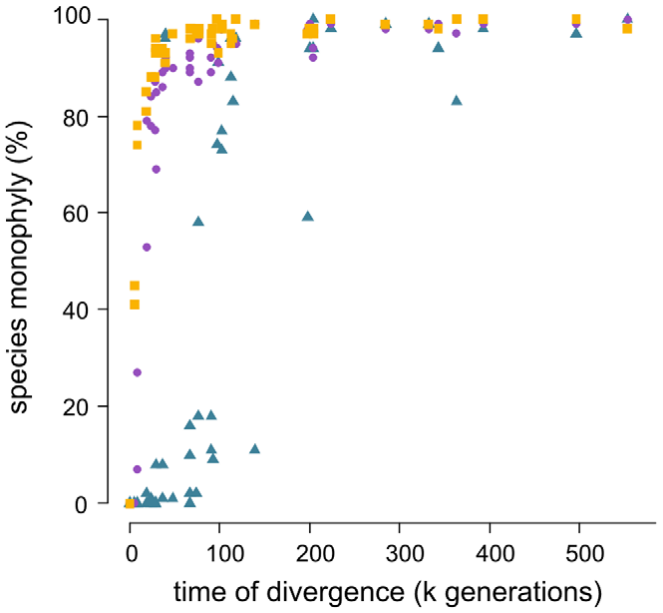
Species monophyly over time of divergence. Scatterplot of percentage species monophyly (N = 100) based on NJ DNA barcode trees for 50 simulated species from the reference data sets (16 individuals per species) plotted against their divergence times. Simulations under coalescence with effective population sizes of 1000 (yellow squares), 10000 (purple dots) and 50000 (blue triangles) individuals.

As expected, percentage of species-monophyly was lower for species that had diverged more recently (Figure 3). While the oldest species (553116 generations) was always monophyletic the two youngest species (98 generations) were never. Between these extremes, percentages increased more rapidly for data sets simulated under coalescence with smaller *N_e_* (Figure 3).

### Method performance

**Figure 4:**
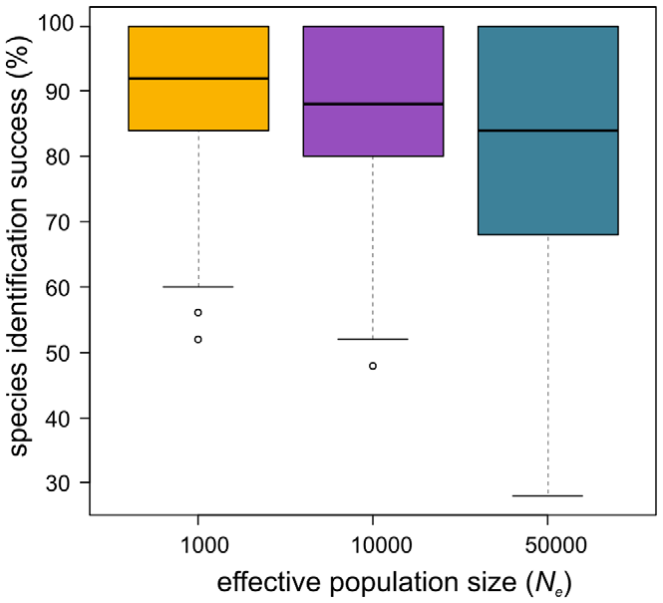
Influence of Effective population size (*N_e_*) on species identification success. Boxplots of percent species identification success (N = 100) based on query data sets simulated under coalescence with effective population sizes of 1000 (yellow), 10000 (purple) and 50000 (blue) individuals.

**Figure 5:**
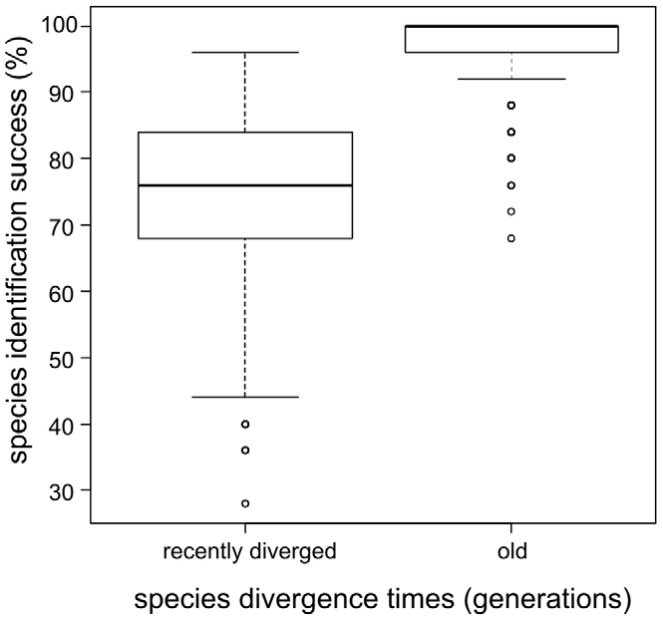
Influence of species divergence on species identification success. Boxplots of percent species identification success (N = 300) based on query data sets for species that were either recently diverged (divergence times between 98 and 76621 generations) or old (divergence times between 76621 and 553116 generations).

The comparative evaluation of methods shows, as expected, that species identification success generally decreased with increasing *N_e_*, see Figure 4 for results across all methods (results for all methods separately are in Table S1). Data sets that were simulated according to the smallest *N_e_* (1,000 individuals) had highest average success score with 89% (P<0.00001). With an average success score of 81%, datasets that were simulated according to the largest *N_e_* (50,000 individuals) were most challenging in terms of species identification (P<0.00001). Similarly, species identification success rates of all methods are lower for species that have diverged more recently, see Figure 5 for results across all methods (results for all methods separately are in Table S2). On average, the 25 recently diverged species were correctly identified in 75% of cases, significantly less than 97% for the 25 old species (P<0.00001). Query identification success showed the same pattern, where scores for old species were generally higher than for recently diverged species and showed less variation (data not shown). We therefore report relative performance of methods compared for recently diverged species only (results for all species are given as supporting information in Table S3 and Figure S2).

**Figure 6:**
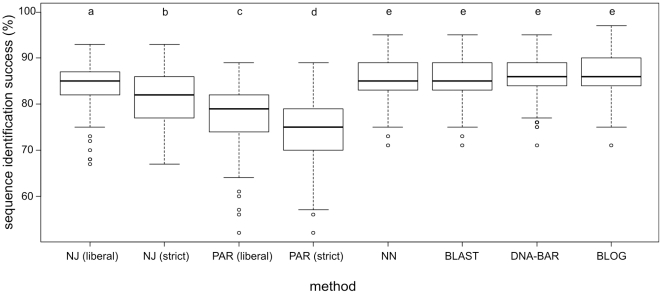
Method performance. Boxplots of sequence identification success (N = 300) of six methods that were applied to recently diverged species in simulated query data sets. NJ  =  neighbor joining, PAR  =  parsimony, NN  =  nearest neighbor. Success scores not significantly different in post-hoc pairwise Wilcoxon tests are indicated by same superscripts.

Diagnostic method BLOG performed best (86.2%) in terms of overall query identification success for recently diverged species based on simulated data (Table 2, Figure 6), although not significant (p = 0.033). Diagnostic method DNA-BAR (86.1%) as well as similarity-based methods NN (85.7%) and BLAST (85.6%) performed only slightly worse than BLOG and significantly better than tree-based methods (P<0.00001). Of the two tree-based methods NJ generally performed better than PAR and liberal assignment performed better than strict assignment for both methods (all P<0.00001).

**Table 2:** Relative method performance based on simulated data for recently diverged species.

Data set	NJ (liberal)	NJ (strict)	PAR (liberal)	PAR (strict)	NN	BLAST	DNA-BAR	BLOG
*N_e_* = 1,000	83.69	83.58	73.31	73.14	86.18	86.18	**86.25**	85.96
*N_e_* = 10,000	85.53	84.27	79.79	78.38	86.11	86.09	86.83	**88.15**
*N_e_* = 50,000	84.20	77.35	79.53	72.32	84.76	84.56	**85.24**	84.58
overall	84.47^a^	81.73^b^	77.54^c^	74.61^d^	85.68^e^	85.61^ e^	86.11^ e^	**86.23^ e^**

DNA barcode query identification success scores (%, N = 100) of six methods applied to barcode sequence datasets simulated under three different effective population sizes (*N_e_*). NJ  =  neighbor joining, PAR  =  parsimony, NN  =  nearest neighbor. Highest scores are in boldface. Overall success scores (%, N = 300) not significantly different in post-hoc pairwise Wilcoxon tests are indicated by same superscripts.

### Empirical data sets

Based on empirical data diagnostic method BLOG performed best (93.1%) in terms of overall query identification success (see Table 3). Diagnostic method DNA-BAR performed only slightly worse (90.4%) and had the best score for two out of three empirical data sets (*Inga* and Cypraeidae). Detailed results per empirical data set can be found in Tables S4, S5, and S6.

**Table 3:** Relative method performance based on empirical data.

Data set	NJ (liberal)	NJ (strict)	PAR (liberal)	PAR (strict)	NN	BLAST	DNA-BAR	BLOG
*Drosophila* (118)	83.90	80.51	83.90	80.51	82.20	83.90	83.90	**96.61**
*Inga* (172)	91.28	90.12	81.40	80.23	88.37	82.56	**94.19**	90.12
Cypraeidae (354)	91.53	90.40	85.31	83.90	91.24	92.66	**93.22**	92.66
Overall	88.90	87.01	83.53	81.55	87.27	86.37	90.43	**93.13**

DNA barcode query identification success scores (%) of six methods applied to three empirical data sets. NJ  =  neighbor joining, PAR  =  parsimony, NN  =  nearest neighbor (liberal)  =  liberal assessment, (strict)  =  strict assessment. Number of query sequences in each data set is in brackets. Overall success scores (bottom line) are averaged over the three data sets. Highest scores are in boldface.

#### *Drosophila*

The most divergent *Drosophila* sequences had 19.5% pairwise distance, and the largest within-species divergence was 17.5% for *D. angor*. Fifteen of 19 species had sufficient coverage (i.e. were represented by 5 or more sequences). Based on the reference data set comprising 497 sequences, 11 species were monophyletic in a NJ tree (73.3%) and 9 had a ‘barcode gap’ (60.0%). Based on the query data set (118 sequences) BLOG outperformed all other methods in terms of query identification success (114 query sequences correctly identified). DNA-BAR and BLAST identified 99 query sequences correctly as did NJ and PAR based on liberal assignment; NN identified 97 query sequences correctly; NJ and PAR identified 95 query sequences correctly based on strict assignment; see Table 3.

#### *Inga*

The two most divergent *Inga* sequences had 1.5% pairwise distance, and largest within-species divergence was 0.7% for *I. capitata*. Thirty five of 56 species had sufficient coverage (i.e. were represented by 5 or more sequences). Based on the reference data set (736 sequences) 25 species were monophyletic in a NJ tree (71.4%) and only 16 had a ‘barcode gap’ (45.7%). Based on the query data set (172 sequences) DNA-BAR outperformed all other methods in terms of query identification success (162 query sequences correctly identified). NJ identified 157 query sequences correctly based on liberal assignment; BLOG identified 155 query sequences correctly as did NJ based on strict assignment. NN identified 152 query sequences correctly; BLAST identified 142 query sequences correctly; PAR identified 140 query sequences correctly based on liberal assignment and 138 based on strict assignment.

#### Cypraeidae

The most divergent Cypraeidae sequences had 28.5% pairwise distance, and largest within-species divergence was 17.1% for *Leporicypraea mappa*. Hundred twelve of 211 species had sufficient coverage (i.e. were represented by 5 or more sequences). Based on the reference data set (1654 sequences) only 81 species were monophyletic in a NJ tree (38.4%) and only 77 had a ‘barcode gap’ (36.5%). Based on the query data set (354 sequences) DNA-BAR outperformed all other methods in terms of query identification success (330 query sequences correctly identified). BLOG and BLAST identified 328 query sequences correctly; NJ identified 324 query sequences correctly based on strict assignment; NN identified 323 query sequences correctly; NJ identified 320 query sequences correctly based on strict assignment; PAR identified 302 query sequences correctly based on liberal assignment and 297 based on strict assignment.

## Discussion

DNA barcoding works well for most species, although significant differences in population dynamics probably exist between, e.g. vertebrates, insects and plants. Indeed, DNA barcoding success rates have been estimated to be around 98% for animals and 70% for plants [96], [97], [98] with the relatively low success rate for the latter having been attributed to various causes such as high incident of hybrid species in angiosperms [99], long generation times or slow mutation rates of woody species [100] and limited dispersal of seeds [100], [101]. Overall, the fact that DNA barcoding works so well is considered to be mainly due to conspecific sequences generally having their coalescent well after time of species divergence [5].

Our results corroborate this notion in that, although our data sets contained incompletely-sorted species, identification success rates were generally high (>80%). Nevertheless, species that are recently diverged pose a consistent problem for identification based on DNA barcodes [1], [2], [31], [32], [33], [34], [39], as indicated by our findings in which methods proved not to be equally robust with regard to incomplete lineage sorting effects in recently diverged species (Figures 3 and 5). As such species are usually of special interest scientifically or from regulatory perspective [1], [2], [3], [12], [13], [14], [27], [28], [29], yet also difficult to identify using morphology [1], [11], [24], [27], [32], [84], finding robust analytical methods is warranted, and commonly used methods such as neighbor joining may not suffice.

### Method performance

#### Tree-based methods

Our results based on simulated data of recently diverged species show that DNA barcode identification of recently diverged species can be significantly improved by applying methods that do not rely on tree representation. The two tree-based methods tested here, i.e. neighbor joining (NJ) and parsimony (PAR), perform worst in terms of query identification success, even with liberal assignment. This finding is in concordance with results from other studies comparing relative performance of DNA barcoding methods [53], [57], [58], [102], as well as with the generally accepted notion that gene trees (i.e. DNA barcode trees) do not necessarily reflect organismal history [42].

PAR consistently and significantly achieved the lowest identification rates here. We see two possible explanations for this result: First, heuristic searches are not guaranteed to find the shortest (i.e. most parsimonious) tree(s) and our search settings may have been insufficiently thorough [88]. Further analysis of some data sets with more thorough search settings did not result in shorter trees being found, however (data not shown), indicating that settings were in fact adequate. Second, several equally parsimonious trees may exist of which only one was used for identification here. Having chosen randomly among equally parsimonious trees may therefore have affected results negatively. NJ will always find a single, fully resolved tree [48] which may have more biological relevance than a randomly chosen maximum parsimony tree, hence resulting in more correct identifications using NJ. We did not include barcode query identification based on a consensus of all most parsimonious trees, but because a consensus tree by definition has reduced resolution we do not expect this could increase performance based on PAR.

For both tree-based methods (i.e. NJ and PAR) strict assignment (i.e. requiring a query to be nested within a monospecific clade for identification) significantly reduced identification success compared to liberal assignment (i.e. allowing identification of a query that is sister to a monospecific clade). This was as expected because when a query is sister to a monospecific clade strict assignment yields an uncertain identification whereas liberal assignment will assign it to the species associated with that clade [37]. Although identification can be wrong in some of these cases, even few correct identifications will result in a higher success rate for liberal assignment compared with strict assignment [37], [57].

There are other tree-based methods for matching DNA barcodes available but we expect that these do not outperform NJ as tested here. For example, Bayesian methods for tree inference [51] do not find a single, fully resolved tree and will therefore share the drawbacks of PAR. The Statistical Assignment Package (SAP) [49] was already found to perform less well than NJ on a Gymnosperm multi-locus DNA barcode data set, even when using the ‘constrained NJ’ algorithm for tree estimation [58].

#### Similarity-based and diagnostic methods

These methods perform significantly better with 31% reduction of error rates compared to tree-based methods (26% when counting tree-based results using liberal assignment only), see Table 2 and Figure 6. Although not significant, diagnostic methods (i.e. BLOG and DNA-BAR) outperformed all other methods tested here. This confirms their suspected superiority as they allow selecting differentiating characters whilst ignoring any obscuring within-species variation [46]. Obviously, diagnostic methods are not guaranteed to have this advantage in all cases. For example, in another study [58] the diagnostic method CAOS [61] did not perform well; possibly because it is dependent upon tree topology for extracting diagnostic characters. The two similarity-based methods (i.e. NN and BLAST) performed only slightly worse compared to the diagnostic methods. This may seem surprising because of the large overlap of within- and between-species distances in our data sets (see Figure 2). But even when there is no ‘barcode gap’ for a particular species, the closest match for a query sequence can well be conspecific, resulting in correct identification [53]. The two methods tested here either require (NN) or produce (BLAST) a sequence alignment, but reliable homology assessment and alignment can be problematic when sequences are variable in length [55], [57]. Alternative similarity-based methods have been proposed that make a projection of sequences based on the decomposition of sequence strings and are therefore in effect alignment-free [35], [55], [56], [57], [58]. String decomposition can be performed in various ways, however, and optimal settings may differ between data sets. For example, preliminary tests of query identification using the recently proposed alignment-free method BRONX [58] showed high success rates for the multi-locus *Inga* data set (90.1%) but very low success rates for the *Drosophila* (53.4%) and Cypraeidae (74.6%) data sets, using the same (default) settings (data not shown).

Although diagnostic and similarity-based methods show similar performance in terms of correct query identification, they markedly differ in their computational cost. Similarity methods such as NN and BLAST are computationally relatively inexpensive because they only involve finding a query’s closest match [37]. By contrast, diagnostic methods must select and extract diagnostic characters, which is computationally expensive [64]. As an example, while the NN analysis of a simulated data set took only ∼2 seconds on a 3GHz dual core desktop computer, analyzing the same data set with BLOG required ∼7 minutes of computation (both analyses using one thread only). Nevertheless, a similarity analysis such as NN has to be repeated for every query sequence requiring identification, thus multiplying the computation time by the number of queries. Diagnostic characters, once they are extracted, can be used to identify any query sequence by simply matching it to these diagnostics – which is much faster than similarity matching in the case of BLOG.

An essential advantage of BLOG over all other methods tested here is that the diagnostic logic formulas extracted by BLOG contain additional information with regards to species identification [64]. Such formulas list the nucleotide(s) by which a species can be differentiated from others and as such can be compared with species descriptions in the traditional taxonomic sense [66]. Other methods can then be compared with trying to match an unknown specimen to all specimens in a collection. We envision that the logic formulas can provide valuable information for other applications. For example, the formulas can be included in species descriptions and taxonomic revisions [103], whereas relative similarities cannot. Obviously, diagnostic formulas exist only relative to a particular alignment but the same is true for morphological characteristics traditionally used for describing species, and in well-sampled clades this problem may well disappear. Diagnostic logic formulas can also be used for designing detection assays based on species-specific nucleotides (e.g. DNA chips and microarrays) and hence assist the development of tools for monitoring and regulation of species. For this purpose DNA-BAR is potentially even better suited than BLOG because it extracts diagnostics that are (combinations of) actual sequence strings that can be used as DNA probes [65]. However, DNA-BAR does not incorporate species-level information in its analysis and selects diagnostics for sequences rather than for species [65]. Moreover, diagnostics selected by DNA-BAR appear to be much more complex than the diagnostic logic formulas extracted by BLOG (personal observations), making DNA-BAR less suitable for extracting species-specific information.

The greatest challenge for diagnostic methods is scalability. Because diagnostic characters are dependent on their context, finding simple diagnostics becomes more difficult with increasing size of the reference database. For example, preliminary analysis of a large data set with 3000 DNA barcodes from over 600 bird species (data not shown) indicate that an alignment of such size is prohibitive for finding simple species-specific logic formulas using the current version of BLOG. Because datasets are ever increasing in size this is an important problem that can be in general tackled in different ways. With reference to this specific application, we see two solutions: 1. A similarity approach with some species groups flagged as ‘problematic’: Identification of a member of such group would then need to be confirmed with diagnostics specific for species in that group. 2. A combined similarity- and diagnostic approach where sequences are first binned into local alignments (e.g. at the level of families or genera) based on similarity; subsequently, diagnostics are applied only within these local alignments.

#### Statistical methods

We did not test any statistical methods for identification based on DNA barcodes. Nevertheless, when species identifications have economic or legal implications (e.g. in detection of quarantine organisms or forensics) there is an obvious need for probabilities associated with barcode matches. However, DNA barcode sequences are essentially short, meaning that they typically contain insufficient information to feed probabilistic models, especially when recently diverged species are concerned. We would therefore advocate confirmation of identifications based on DNA barcodes by other lines of evidence (e.g. multiple independent loci, serological tests or morphological expert opinion) rather than relying on DNA barcodes only in such cases.

### Empirical data sets

Our results based on empirical data are largely consistent with results based on simulated data. Few differences in overall results exist, however: Where scores for tree-based NJ were suboptimal based on simulated data, they were comparable to at least some of the similarity-based and diagnostic methods when applied to the empirical data sets. For the *Drosophila* data set PAR performed equally well as NJ. It should be noted that with only three data sets assessing significance of differences in method performance is limited, underlining the advantage of using simulated data. In addition, DNA barcode identification success can depend on taxonomic sampling. In ‘regional’ data sets (i.e. samples from a particular geographic region only) within-species variation is usually underestimated because of un-sampled haplotypes, while between-species differences are usually overestimated because of un-sampled taxa [31], [38], [39], [53], [104]. Therefore, regional data sets such as *Inga* are expected to inflate DNA barcode identification success rates in contrast to ‘clade-based’ data sets (i.e. sampling all extant species across their entire distribution) such as Cypraeidae. Nevertheless, because the selected data sets comprise genetic markers from all three genomic compartments, result from different sampling efforts and represent broad phylogenetic diversity (i.e. insects, plants and gastropods) we interpret consistency in our findings as an indication that they will equally apply to other genetic markers and clades.

### Conclusion

We found similarity-based (NN, BLAST) and diagnostic methods (BLOG, DNA-BAR) to significantly outperform tree-based methods (NJ, PAR), when applied to simulated DNA barcode data of recently diverged species. Diagnostic methods BLOG and DNA-BAR performed best on both simulated and empirical data and BLOG had the highest correct query identification rate overall. Although similarity-based methods have better scalability compared to BLOG they do not reveal any species-level information that can be used outside the realm of DNA barcoding. Diagnostic logic formulas extracted by BLOG provide information that can be used for e.g. taxonomy and species detection assays. Method choice therefore should depend on requirement of either computation speed or information content. In the end, recently diverged species remain difficult to identify, but we expect that our results contribute to alleviating this problem.

## Supporting Information

Figure S1 **Simulated ultrametric species tree.** Tree with 50 species simulated under the Yule model and with a total tree depth of 1 million generations. Terminal branches subtending species considered as ‘recently diverged’ are in red, those subtending species considered as ‘old’ are in blue.
(TIF)

Figure S2 **Relative method performance based on simulated data for all species.** Boxplots of query identification success (N = 300) of six methods that were applied to ‘recently diverged’ species in simulated query data sets. NJ  =  neighbor joining, PAR  =  parsimony, NN  =  nearest neighbor. Success scores not significantly different in post-hoc pairwise Wilcoxon tests are indicated by same superscripts.
(TIF)

Table S1 **Influence of effective population size (** ***N_e_*** **) on species identification success per method compared.**
(PDF)

Table S2 **Influence of divergence time on species identification success per method compared.**
(PDF)

Table S3 **Method performance based on simulated data for all species.**
(PDF)

Table S4 **Results for all 15 species represented by 5 or more sequences in the** ***Drosophila*** **empirical data set.**
(PDF)

Table S5 **Results for all 35 species represented by 5 or more sequences in the** ***Inga*** **empirical data set.**
(PDF)

Table S6 **Results for all 112 species represented by 5 or more sequences in the Cypraeidae empirical data set.**
(PDF)
