# Supplementary figures and images for: DNA Barcoding of Recently Diverged Species: Relative Performance of Matching Methods
Source: PLoS One. 2012 Jan 17;7(1):e30490. doi: 10.1371/journal.pone.0030490 (PMC3260286; doi:10.1371/journal.pone.0030490)

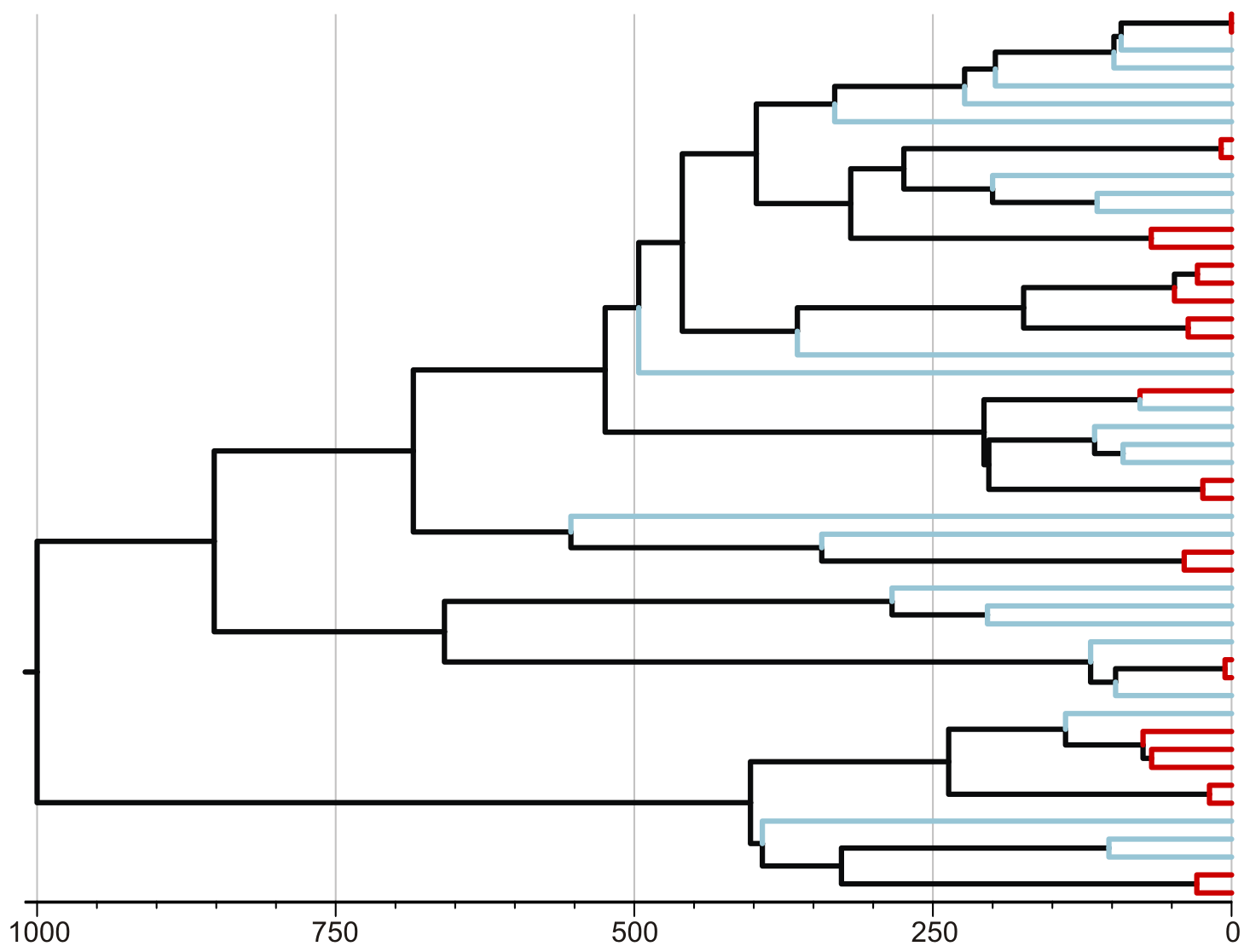

Supplement: Figure S1 — Simulated ultrametric species tree. Tree with 50 species simulated under the Yule model and with a total tree depth of 1 million generations. Terminal branches subtending species considered as ‘recently diverged’ are in red, those subtending species considered as ‘old’ are in blue. (TIF) [file pone.0030490.s001.tif]

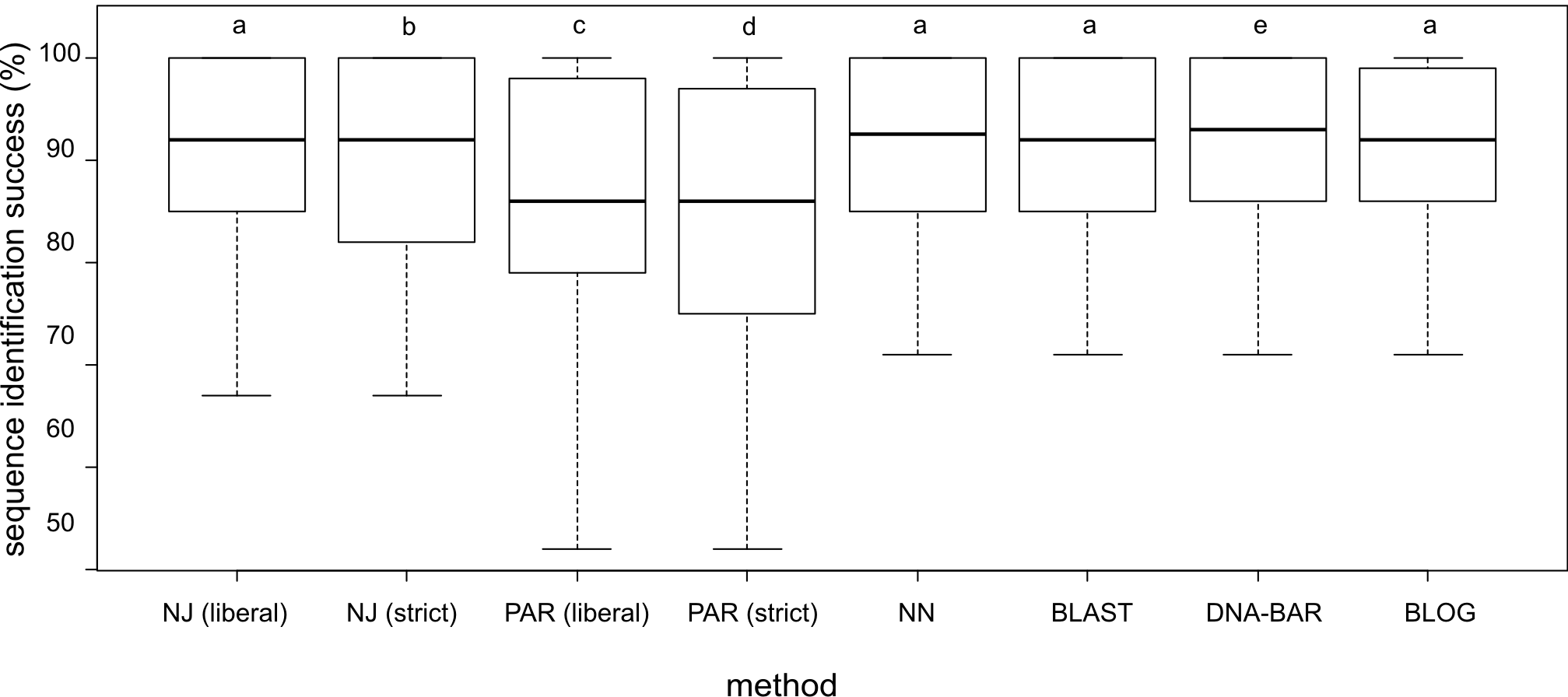

Supplement: Figure S2 — Relative method performance based on simulated data for all species. Boxplots of query identification success (N = 300) of six methods that were applied to ‘recently diverged’ species in simulated query data sets. NJ = neighbor joining, PAR = parsimony, NN = nearest neighbor. Success scores not significantly different in post-hoc pairwise Wilcoxon tests are indicated by same superscripts. (TIF) [file pone.0030490.s002.tif]
