# Supplementary material for: DNA Barcoding of Recently Diverged Species: Relative Performance of Matching Methods
Source: PLoS One. 2012 Jan 17;7(1):e30490. doi: 10.1371/journal.pone.0030490 (PMC3260286; doi:10.1371/journal.pone.0030490)
Supplement: Table S1 — Influence of effective population size ( Ne ) on species identification success per method compared. (PDF) [file pone.0030490.s003.pdf]

Table S1. Influence of effective population size (N_e) on species identification success per method compared

Data set	NJ (liberal)	NJ (strict)	PAR (liberal)	PAR (strict)	NN	BLAST	DNA-BAR	BLOG
Ne = 1000	90.4	90.4	82.7	82.7	92.1	92.1	**92.1**	91.9
Ne = 10000	88.9	88.2	82.8	82.2	89.8	89.8	90.1	**90.2**
Ne = 50000	84.2	79.0	78.5	73.7	85.7	85.4	**85.8**	78.1

Species identification success scores (%, N=100) of six methods applied to query data sets simulated under three different effective population sizes (*Ne*). NJ = neighbor joining, PAR = parsimony, NN = nearest neighbor.
