# Supplementary material for: DNA Barcoding of Recently Diverged Species: Relative Performance of Matching Methods
Source: PLoS One. 2012 Jan 17;7(1):e30490. doi: 10.1371/journal.pone.0030490 (PMC3260286; doi:10.1371/journal.pone.0030490)
Supplement: Table S2 — Influence of divergence time on species identification success per method compared. (PDF) [file pone.0030490.s004.pdf]

Table S2. Influence of divergence time on species identification success per method compared

Species divergence time	NJ (liberal)	NJ (strict)	PAR (liberal)	PAR (strict)	NN	BLAST	DNA-BAR	BLOG
old	99.2	99.1	96.9	96.6	99.4	99.4	99.5	98.0
recent	84.5	81.7	77.5	74.6	85.7	85.6	86.1	86.2

Species identification success scores (%, N=300) based on query data sets for species that were either recently diverged (divergence times between 98 and 76,621 generations) or old (divergence times between 76,621 and 553,116 generations). NJ = neighbor joining, PAR = parsimony, NN = nearest neighbor.
