# Supplementary material for: DNA Barcoding of Recently Diverged Species: Relative Performance of Matching Methods
Source: PLoS One. 2012 Jan 17;7(1):e30490. doi: 10.1371/journal.pone.0030490 (PMC3260286; doi:10.1371/journal.pone.0030490)
Supplement: Table S3 — Method performance based on simulated data for all species. (PDF) [file pone.0030490.s005.pdf]

Table S3. Method performance based on simulated data for all species

Data set	NJ (liberal)	NJ (strict)	PAR (liberal)	PAR (strict)	NN	BLAST	DNA-BAR	BLOG
Ne = 1000	91.06	91.00	83.89	83.79	92.54	92.54	**92.57**	92.36
Ne = 10000	92.47	91.81	88.55	87.75	92.86	92.85	93.23	**93.60**
Ne = 50000	91.99	88.42	89.22	85.29	92.30	92.19	**92.54**	90.39
overall	91.84[a]	90.41[b]	87.22[c]	85.61[d]	92.56[a]	92.52[a]	**92.78[e]**	92.11[a]

DNA barcode query sequence identification success scores (%, N=100) of six methods applied to query data sets simulated under three different effective population sizes (*Ne*). NJ = neighbor joining, PAR = parsimony, NN = nearest neighbor. Highest scores are in boldface. Overall success scores (%, N=300) not significantly different in post-hoc pairwise Wilcoxon tests are indicated by same superscripts.
