# Supplementary material for: DNA Barcoding of Recently Diverged Species: Relative Performance of Matching Methods
Source: PLoS One. 2012 Jan 17;7(1):e30490. doi: 10.1371/journal.pone.0030490 (PMC3260286; doi:10.1371/journal.pone.0030490)
Supplement: Table S4 — Results for all 15 species represented by 5 or more sequences in the Drosophila empirical data set. (PDF) [file pone.0030490.s006.pdf]

Table S4. Results for all 15 species represented by 5 or more sequences in the *Drosophila* empirical data set

species	monophyly	barcode gap	#refs	#qrs	NJ (liberal)	NJ (strict)	PAR (liberal)	PAR (strict)	NN	BLAST	DNA-BAR	BLOG
D. angor	FALSE	FALSE	10	2	2	2	2	2	2	2	1	2
D. arizonae	TRUE	FALSE	14	3	3	3	3	3	3	3	3	3
D. barutani	TRUE	TRUE	5	1	1	1	1	1	1	1	1	1
D. falleni	TRUE	TRUE	12	3	3	3	3	3	3	3	3	3
D. innubila	TRUE	TRUE	23	6	6	6	6	6	6	6	6	6
D. melanogaster	TRUE	TRUE	8	2	2	2	2	2	2	2	2	2
D. mettleri	TRUE	TRUE	19	5	5	5	5	5	5	5	5	4
D. mojavensis	TRUE	FALSE	38	9	9	9	9	9	9	9	9	8
D. montana	TRUE	TRUE	34	8	8	8	8	8	8	8	8	7
D. nigrospiracula	TRUE	TRUE	8	2	2	2	2	2	2	2	2	2
D. pachea	TRUE	TRUE	63	16	16	16	16	16	16	16	16	16
D. recens	FALSE	FALSE	109	27	9	5	9	5	7	9	10	27
D. simulans	FALSE	FALSE	22	5	5	5	5	5	5	5	5	5
D. subquinaria	FALSE	FALSE	109	27	26	26	26	26	26	26	26	26
D. virilis	TRUE	TRUE	9	2	2	2	2	2	2	2	2	2
Overall	11	9	483	118	99	95	99	95	97	99	99	**114**

List of species names, species monophyly and barcode gap, number of sequences, and sequence identification success scores. #refs = number of sequences in the reference data set; #qrs= number of sequences in the query data set; NJ = neighbor joining, PAR = parsimony, NN = nearest neighbor. Highest overall score is in boldface.
