# Supplementary material for: DNA Barcoding of Recently Diverged Species: Relative Performance of Matching Methods
Source: PLoS One. 2012 Jan 17;7(1):e30490. doi: 10.1371/journal.pone.0030490 (PMC3260286; doi:10.1371/journal.pone.0030490)
Supplement: Table S5 — Results for all 35 species represented by 5 or more sequences in the Inga empirical data set. (PDF) [file pone.0030490.s007.pdf]

Table S5. Results for all 35 species represented by 5 or more sequences in the *Inga* empirical data set

Species	monophyly	barcode gap	#refs	#qrs	NJ (liberal)	NJ (strict)	PAR (liberal)	PAR (strict)	NN	BLAST	DNA-BAR	BLOG
I. acreana	TRUE	FALSE	22	6	5	5	5	5	5	5	5	5
I. alata	TRUE	TRUE	46	12	12	12	12	12	12	0	12	12
I. alba	TRUE	TRUE	33	8	8	8	8	8	8	8	8	8
I. auristellae	TRUE	TRUE	48	12	11	11	12	12	12	9	11	11
I. bourgonii	FALSE	FALSE	62	15	14	14	8	7	14	14	15	14
I. capitata	FALSE	FALSE	20	5	5	5	5	5	5	5	5	5
I. cayennensis	FALSE	FALSE	12	3	0	0	0	0	0	0	3	0
I. chartacea	FALSE	FALSE	11	3	3	3	3	3	3	3	3	3
I. cinnamomea	TRUE	TRUE	15	4	4	4	4	4	4	4	4	4
I. coruscans	TRUE	FALSE	8	2	2	2	0	0	0	2	2	2
I. densiflora	FALSE	FALSE	7	2	2	1	1	1	2	1	2	2
I. edulis	TRUE	FALSE	36	9	9	9	9	9	9	9	9	9
I. fosteriana	TRUE	TRUE	6	1	1	1	1	1	1	1	1	1
I. heterophylla	TRUE	TRUE	4	1	1	1	1	1	1	1	1	1
I. laurina	TRUE	FALSE	22	6	6	6	6	6	6	6	6	6
I. leiocalycina	FALSE	FALSE	23	6	5	5	5	5	6	5	6	4
I. marginata	TRUE	FALSE	42	10	10	10	10	10	10	10	10	10
I. morphosp17	FALSE	FALSE	12	3	3	3	3	3	3	3	3	2
I. morphosp18	TRUE	FALSE	6	2	2	2	0	0	1	2	2	2
I. morphosp22	TRUE	TRUE	6	1	1	1	1	1	1	1	1	1
I. morphosp49	TRUE	TRUE	4	1	1	1	1	1	1	1	1	1
I. morphosp50	TRUE	TRUE	4	1	1	1	1	1	1	1	1	1
I. morphosp75	TRUE	TRUE	9	2	2	2	2	2	2	2	2	2

I. nobilis	TRUE	TRUE	7	2	2	2	2	2	2	2	2	2
I. poeppigiana	TRUE	TRUE	10	3	3	3	3	3	3	3	3	3
I. porcata	TRUE	TRUE	11	3	3	3	3	3	3	3	3	3
I. punctata	TRUE	FALSE	18	4	4	4	0	0	4	4	4	4
I. ruiziana	TRUE	TRUE	40	10	10	10	10	10	10	10	10	10
I. sapindoides	FALSE	FALSE	31	8	8	8	8	8	8	8	8	8
I. stipulacea	FALSE	FALSE	4	1	1	0	1	0	1	1	1	1
I. suaveolans	TRUE	FALSE	18	4	4	4	4	4	0	4	4	4
I. thibaudiana	FALSE	FALSE	32	8	0	0	0	0	0	0	0	0
I. tomentosa	TRUE	TRUE	5	1	1	1	1	1	1	1	1	1
I. umbellifera	TRUE	TRUE	40	10	10	10	10	10	10	10	10	10
I. venusta	TRUE	FALSE	11	3	3	3	0	0	3	3	3	3
Overall	25	16	685	172	157	155	140	138	152	142	**162**	155

List of species names, species monophyly and ‘barcode gap’, number of sequences, and sequence identification success scores. #refs = number of sequences in the reference data set; #qrs= number of sequences in the query data set; NJ = neighbor joining, PAR = parsimony, NN = nearest neighbor. Highest overall score is in boldface.
