# Supplementary material for: DNA Barcoding of Recently Diverged Species: Relative Performance of Matching Methods
Source: PLoS One. 2012 Jan 17;7(1):e30490. doi: 10.1371/journal.pone.0030490 (PMC3260286; doi:10.1371/journal.pone.0030490)
Supplement: Table S6 — Results for all 112 species represented by 5 or more sequences in the Cypraeidae empirical data set. (PDF) [file pone.0030490.s008.pdf]

Table S6. Results for all 112 species represented by 5 or more sequences in the Cypraeidae empirical data set

	monophyly	barcode gap	#refs	#qrs	NJ (liberal)	NJ (strict)	PAR (liberal)	PAR (strict)	NN	BLAST	DNA-BAR	BLOG
Annepona_mariae	TRUE	TRUE	4	1	1	1	1	1	1	1	1	1
Bistolia_hirundo	TRUE	TRUE	10	3	3	3	3	3	3	3	3	3
Bistolida_kieneri	TRUE	FALSE	13	3	3	3	3	3	3	3	3	3
Bistolida_stolida	FALSE	FALSE	14	4	4	4	4	4	4	4	4	4
Blasicrura_pallidula	FALSE	FALSE	5	1	1	1	1	1	1	1	1	1
Chelycypraea_testudinaria	TRUE	TRUE	4	1	1	1	1	1	1	1	1	1
Contradusta_walkeri	TRUE	TRUE	5	1	1	1	1	1	1	1	1	0
Cribrarula_cribraria	FALSE	FALSE	51	13	13	13	13	12	13	13	13	13
Cribrarula_cumingii	FALSE	FALSE	18	4	4	2	2	2	2	4	4	4
Cribrarula_esontropia	TRUE	TRUE	5	1	1	1	1	0	1	1	1	1
Cribrarula_exmouthensis	FALSE	FALSE	5	1	1	1	1	1	1	1	1	1
Cribrarula_gaskoinii	TRUE	TRUE	9	2	2	2	2	2	2	2	2	2
Cypraea_tigris	FALSE	FALSE	62	15	15	15	15	15	15	15	15	15
Eclogavena_dayritiana	TRUE	TRUE	4	1	1	1	1	1	1	1	1	1
E._quadrimaculata	FALSE	FALSE	6	2	2	2	1	1	2	2	2	2
Erosaria_acicularis	TRUE	TRUE	4	1	1	1	1	1	1	1	1	1
Erosaria_beckii	FALSE	FALSE	4	1	1	1	1	1	1	1	1	1
Erosaria_boivinii	TRUE	TRUE	4	1	1	1	1	1	1	1	1	1
Erosaria_cernica	TRUE	TRUE	6	1	1	1	1	1	1	1	1	1
Erosaria_englerti	TRUE	TRUE	5	1	1	1	1	1	1	1	1	1
Erosaria_erosa	TRUE	TRUE	13	3	3	3	3	3	3	3	3	3
Erosaria_helvola	TRUE	TRUE	71	18	18	18	18	18	18	18	18	18
Erosaria_labrolineata	TRUE	TRUE	4	1	1	1	1	1	1	1	1	1

Erosaria_lamarckii	TRUE	TRUE	8	2	2	2	2	2	2	2	2	2
Erosaria_nebrites	TRUE	TRUE	4	1	1	1	1	1	1	1	1	1
Erosaria_ocellata	TRUE	TRUE	4	1	1	1	1	1	1	1	1	1
Erosaria_poraria	TRUE	TRUE	4	1	1	1	1	1	1	1	1	1
Erosaria_spurca	FALSE	FALSE	5	1	1	1	1	1	1	1	1	1
Erosaria_thomasi	TRUE	TRUE	4	1	1	1	1	1	1	1	1	1
Erosaria_turdus	TRUE	TRUE	5	1	1	1	1	1	1	1	1	1
Erronea_caurica	FALSE	FALSE	72	18	18	17	15	15	18	18	18	18
Erronea_cylindrica	FALSE	FALSE	14	4	4	4	2	1	4	4	4	4
Erronea_errones	FALSE	FALSE	28	7	5	5	5	5	5	5	5	3
Erronea_onyx	TRUE	TRUE	10	2	1	1	1	1	1	1	1	1
Erronea_ovum	FALSE	FALSE	26	6	6	6	6	6	6	6	6	5
Erronea_subviridis	TRUE	TRUE	6	2	2	2	2	2	2	2	2	2
Erronea_succincta	TRUE	TRUE	4	1	1	1	1	1	1	1	1	1
Erronea_vredenburgi	TRUE	TRUE	4	1	1	1	1	1	1	1	1	1
Leporicypraea_geographica	TRUE	TRUE	14	4	4	4	4	4	4	4	4	3
Leporicypraea_mappa	FALSE	FALSE	32	8	8	8	8	8	8	8	8	8
Luria_cinerea	TRUE	TRUE	5	1	1	1	1	1	1	1	1	0
Luria_isabella	FALSE	FALSE	12	3	3	3	0	0	3	3	3	3
Luria_lurida	TRUE	TRUE	5	1	1	1	1	1	1	1	1	1
Luria_tessellata	TRUE	TRUE	4	1	1	1	1	1	1	1	1	0
Lyncina_carneola	TRUE	TRUE	17	4	4	4	4	4	4	4	4	4
Lyncina_leviathan	TRUE	TRUE	5	1	1	1	1	1	1	1	1	1
Lyncina_lynx	TRUE	TRUE	4	1	1	1	1	1	1	1	1	1
Lyncina_schilderorum	TRUE	TRUE	5	1	1	1	1	1	1	1	1	1
Lyncina_ventriculus	TRUE	TRUE	6	1	1	1	1	1	1	1	1	1

Lyncina_vitellus	TRUE	TRUE	5	1	1	1	1	1	1	1	1	1
Macrocypraea_cervinetta	TRUE	TRUE	4	1	1	1	1	1	1	1	1	1
Macrocypraea_zebra	FALSE	FALSE	6	1	0	0	0	0	0	0	1	1
Mauritia_arabica	TRUE	FALSE	110	27	27	27	27	27	27	27	27	27
Mauritia_depressa	TRUE	TRUE	18	4	4	4	4	4	4	4	4	4
Mauritia_eglantina	TRUE	TRUE	5	1	1	1	1	1	1	1	1	1
Mauritia_grayana	TRUE	TRUE	10	3	3	3	3	3	3	3	3	3
Mauritia_histrio	TRUE	TRUE	5	1	1	1	1	1	1	1	1	1
Mauritia_maculifera	TRUE	TRUE	41	10	10	10	10	10	10	10	10	10
Mauritia_mauritiana	TRUE	TRUE	5	1	1	1	1	1	1	1	1	1
Mauritia_scurra	FALSE	FALSE	10	2	2	2	2	2	2	2	2	2
Melicerona_felina	TRUE	FALSE	5	1	1	1	1	1	1	1	1	1
Melicerona_listeri	FALSE	FALSE	8	2	2	2	1	1	2	2	2	2
Monetaria_annulus	FALSE	FALSE	52	13	8	7	3	1	10	10	10	11
Monetaria_caputdraconis	TRUE	TRUE	6	2	2	2	2	2	2	2	2	2
Monetaria_caputserpentis	FALSE	FALSE	20	5	5	5	3	3	5	5	5	5
Monetaria_moneta	TRUE	TRUE	8	2	2	2	2	2	2	2	2	2
Monetaria_obvelata	FALSE	FALSE	12	3	1	1	1	1	1	1	1	0
Neobernaya_spadicea	TRUE	TRUE	4	1	1	1	1	1	1	1	1	1
Notadusta_punctata	TRUE	FALSE	34	9	9	9	9	9	9	9	9	9
Notocypraea_angustata	TRUE	TRUE	6	1	1	1	1	1	1	1	1	1
Notocypraea_comptoni	TRUE	TRUE	14	4	4	4	4	4	4	4	4	4
Notocypraea_declivis	TRUE	TRUE	5	1	1	1	1	1	1	1	1	1
Notocypraea_piperita	FALSE	FALSE	9	2	1	1	0	0	1	1	1	2
Nucleolaria_nucleus	FALSE	FALSE	7	2	2	2	2	2	2	2	2	2
Ovatipsa_chinensis	TRUE	TRUE	14	3	3	3	3	3	3	3	3	3

Ovatipsa_coloba	TRUE	TRUE	6	1	1	1	1	1	1	1	1	1
Palmadusta_artuffeli	TRUE	TRUE	6	2	2	2	2	2	2	2	2	2
Palmadusta_asellus	TRUE	TRUE	7	2	2	2	2	2	2	2	2	2
Palmadusta_clandestina	FALSE	FALSE	22	5	5	5	5	5	5	5	5	5
Palmadusta_contaminata	TRUE	TRUE	5	1	1	1	1	1	1	1	1	1
Palmadusta_lentiginosa	TRUE	TRUE	4	1	1	1	1	1	1	1	1	1
Postularia_margarita	TRUE	TRUE	4	1	1	1	1	1	1	1	1	1
Pseudozonaria_robertsi	TRUE	TRUE	4	1	1	1	1	1	1	1	1	1
Purpuradusta_fimbriata	TRUE	TRUE	49	12	12	12	12	12	12	12	12	12
Purpuradusta_gracilis	TRUE	TRUE	13	3	3	3	3	3	3	3	3	3
Purpuradusta_hammondae	TRUE	TRUE	4	1	1	1	1	1	1	1	1	1
Purpuradusta_microdon	TRUE	TRUE	5	1	1	1	1	1	1	1	1	0
Purpuradusta_minoridens	TRUE	TRUE	6	1	1	1	1	1	1	1	1	1
Purpuradusta_oryzaeformis	TRUE	TRUE	4	1	1	1	1	1	1	1	1	1
Pustularia_bistrinotata	TRUE	TRUE	9	2	2	2	2	2	2	2	2	2
Pustularia_cicercula	TRUE	TRUE	9	2	2	2	2	2	2	2	2	2
Pustularia_globulus	TRUE	TRUE	6	1	1	1	1	1	1	1	1	1
Pustularia_mauiensis	TRUE	TRUE	5	1	1	1	1	1	1	1	1	1
Staphylaea_limacina	TRUE	TRUE	8	2	2	2	2	2	2	2	2	2
Staphylaea_staphylaea	TRUE	TRUE	10	2	2	2	2	2	2	2	2	2
Talostolida_pellucens	TRUE	TRUE	22	5	5	5	5	5	5	5	5	5
Talostolida_subteres	TRUE	TRUE	5	1	1	1	1	1	1	1	1	1
Talostolida_teres	TRUE	TRUE	9	2	2	2	2	2	2	2	2	2
Trona_stercoraria	TRUE	TRUE	4	1	1	1	1	1	1	1	1	1
Umbilia_armeniaca	TRUE	TRUE	8	2	2	2	2	2	2	2	2	2
Umbilia_capricornica	TRUE	TRUE	4	1	1	1	1	1	1	1	1	1

Umbilia_hesitata	TRUE	TRUE	9	2	2	2	2	2	2	2	2	2
Zoila_decipiens	FALSE	FALSE	12	3	1	1	1	1	1	1	1	1
Zoila_friendii	FALSE	FALSE	41	10	0	0	0	0	0	3	3	9
Zoila_jeaniana	FALSE	FALSE	23	6	6	6	6	6	6	6	6	6
Zoila_ketyana	FALSE	FALSE	18	4	2	2	2	2	1	1	2	4
Zoila_marginata	FALSE	FALSE	14	3	3	3	1	1	3	3	3	0
Zoila_perlae	FALSE	FALSE	9	2	0	0	0	0	0	0	0	0
Zoila_rosselli	TRUE	TRUE	13	3	3	3	3	3	3	3	3	3
Zoila_thersites	FALSE	FALSE	10	2	0	0	0	0	0	0	0	0
Zoila_venusta	TRUE	TRUE	18	5	5	5	5	5	5	5	5	5
Zonaria_zonaria	TRUE	TRUE	4	1	1	1	1	1	1	1	1	1
Overall	81	77	1465	354	324	320	302	297	323	328	**330**	328

List of species names, species monophyly and ‘barcode gap’, number of sequences, and sequence identification success scores. #refs = number of sequences in the reference data set; #qrs= number of sequences in the query data set; NJ = neighbor joining, PAR = parsimony, NN = nearest neighbor. Highest overall score is in boldface.
